# Mapping with Monocular Camera Sensor under Adversarial Illumination for Intelligent Vehicles

**DOI:** 10.3390/s23063296

**Published:** 2023-03-21

**Authors:** Wei Tian, Yongkun Wen, Xinning Chu

**Affiliations:** School of Automotive Studies, Tongji University, Shanghai 201804, China

**Keywords:** intelligent vehicle, monocular camera sensor, visual mapping, adversarial illumination, unsupervised keypoint learning, scale drift reduction

## Abstract

High-precision maps are widely applied in intelligent-driving vehicles for localization and planning tasks. The vision sensor, especially monocular cameras, has become favoured in mapping approaches due to its high flexibility and low cost. However, monocular visual mapping suffers from great performance degradation in adversarial illumination environments such as on low-light roads or in underground spaces. To address this issue, in this paper, we first introduce an unsupervised learning approach to improve keypoint detection and description on monocular camera images. By emphasizing the consistency between feature points in the learning loss, visual features in dim environment can be better extracted. Second, to suppress the scale drift in monocular visual mapping, a robust loop-closure detection scheme is presented, which integrates both feature-point verification and multi-grained image similarity measurements. With experiments on public benchmarks, our keypoint detection approach is proven robust against varied illumination. With scenario tests including both underground and on-road driving, we demonstrate that our approach is able to reduce the scale drift in reconstructing the scene and achieve a mapping accuracy gain of up to 0.14 m in textureless or low-illumination environments.

## 1. Introduction

The advent of big data and Internet of Things (IoT) has brought new prospects for intelligent vehicles [1,2,3,4]. A high-precision map is a prerequisite for the localization and planning of intelligent vehicles, and its creation is considered a key technology in this field and is well-researched with various sensor setups. For instance, Global Navigation Satellite System (GNSS)-based devices are integrated in many commercial vehicles. However, the satellite signal can be easily shielded by high buildings or specific materials. Lidar can directly provide accurate distance information and is thus widely used in mapping approaches. Yet the high resolution of 3D representation is at the cost of high data volume and non-negligible hardware cost. On the contrary, vision-based solutions, especially with monocular images, benefiting from the low sensor cost and effectiveness in texture-rich scenarios [5], have attracted much attention in mapping research.

Traditional visual mapping is based on the structure from motion (SfM) paradigm. It extracts many keypoints with feature descriptors from images captured in a scene. The 3D positions of these keypoints are either provided using additional measuring instruments or triangulated with multi-view geometry. These keypoints are stored and further utilized as landmarks to construct geometrical constraints for ego-pose estimation in vehicle localization tasks. In this way, the output of visual mapping can be presented with a point cloud consisting of sparsely distributed 3D keypoints along with their descriptors.

**Figure 1:**
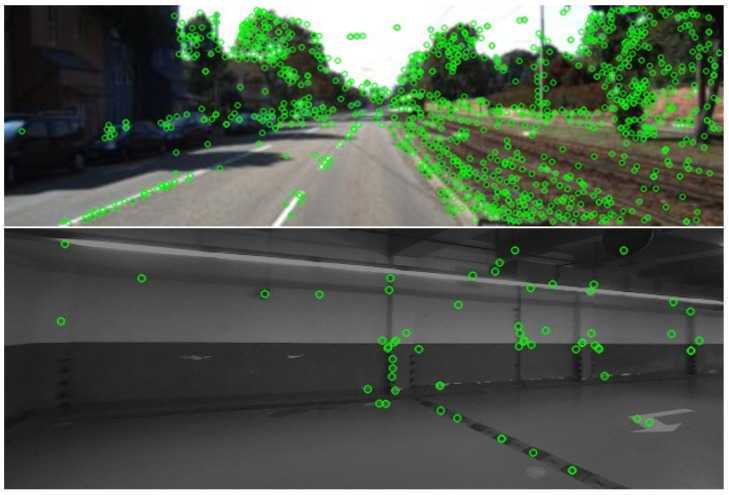
Detected SIFT keypoints (green circles) in ordinary outdoor scenario (**above**) and dim textureless garage (**below**).

However, monocular visual mapping approaches are known to have a relatively low capacity in accurately estimating the depth information and the absolute scale. Moreover, handcrafed keypoint models such as SIFT [6], SURF [7], ORB [8], BRISK [9], etc., are prone to feature-extraction failure in low-texture and low-illumination environments such as underground spaces, where the keypoint detection and matching performance significantly degrades (Figure 1), further deteriorating the generated maps. Many researchers have been working on these problems, especially leveraging the powerful perception capability of the rising deep learning technology. For instance, the semantic features are considered as landmarks in the image [10] and also adopted to improve matching in low-textured environments such as in underground garages [11]. Features such as simple visual tags are used to reduce the computational costs and improve the location accuracy [12]. Furthermore, high-level landmarks such as visual fiducial markers are simultaneously perceived to aid the pose-estimation process [13]. In other attempts, keypoint detection and description are learned by convolutional neural networks (CNNs) to replace handcrafted ORB keypoints and integrated into simultaneous localization and mapping (SLAM) systems [14]. Nevertheless, the segmentation model requires a large amount of pixel-wise labeled image data for training, and the setup of fiducial markers should be elaborately designed, which limits the generalizability of the mapping approach. Moreover, the groundtruths (GTs) of keypoints in [14] are provided by the Shi–Tomasi detector [15]. In comparison to CNN-based methods, which can extract complex visual features [16], approaches that learn from such a traditional keypoint-detection paradigm can be suboptimal in scenarios with less texture.

To address these issues, this paper presents a monocular visual mapping approach in low-textured and illumination-changed environments using unsupervised keypoint learning and improved scale recovery (codes will be available at https://github.com/XinningC/Adversarial_mono_Mapping (accessed on 18 February 2023)). Our approach comprises the following two components: unsupervised keypoint extraction and 3D reconstruction with improved scale estimation. According to the experimental results of keypoint extraction and mapping in Section 4, we show that our approach outperforms existing mainstream methods with an average location error gap of 0.04 m on Euroc [17] and 1.86 m on KITTI [18] and manifests robustness in adversarial-illumination environments. Our contributions are as follows:

- We introduce an unsupervised keypoint-detection and -description approach into the visual mapping process with an improved loss to enhance the learning of discriminative descriptors. Such an approach only requires monocular images as input, thus saving annotation labors.
- We present a scheme integrating both feature-point verification and self-supervised multi-grained image similarity measurements. It effectively reduces the cumulative error and suppresses the overall scale drift at a low level.
- We further integrate ground point features and camera height to recover the absolute scale. Using validation on both the self-collected data and public benchmarks, this mapping approach is demonstrated to be robust against illumination changes in scenarios such as underground parking and outdoor roads.

The remainder of this paper is organized as follows. An overview of the related work is given in Section 2. Section 3 introduces the proposed monocular visual mapping approach using unsupervised keypoint matching and improved scale recovery. Section 4 evaluates the mapping in both underground parking and outdoor driving. The conclusion is given in Section 5.

## 2. Related Work

In this section, existing studies are reviewed in three aspects mostly related to the proposed approach: (1) keypoint models; (2) monocular visual architecture; (3) mapping under adversarial illumination.

### 2.1. Keypoint Models

Traditional visual mapping approaches are based on handcrafted keypoint models, including SIFT [6], SURF [7], ORB [8], BRISK [9], etc. SIFT preserves a good invariance to rotation and scaling by leveraging gradient histograms, but its computational complexity is high. Although improvements such as the approximated gradient filtering are adopted in SURF, the runtime gain is still limited. Unlike SURF, ORB achieves an excellent real-time performance by employing the FAST keypoint detector [19] and the BRIEF feature descriptor [20]. Since feature points are extracted by simple comparison with surrounding pixels, the reliability in illumination-changed and low-textured environments is relatively poor. ASIFT [21] outperforms SIFT while features are undergoing large transition tilt due to its affinity invariance; nevertheless, the matching accuracy and efficiency need improvement [22,23]. BRISK employs feature pyramid and Gaussian filtering for point sampling, achieving an improved noise robustness, but at the cost of increased computation amount.

In recent years, deep-learning-based feature-point methods have gained much attention. Lift [24] proposes a full feature-point handling pipeline, including point detection, orientation estimation, and feature description, while a supervision form SfM system is required. Superpoint [25] proposes an unsupervised learning for feature extraction with a fully convolutional neural network (FCN) and a homography adaption, but it relies on labeled interest-point images for pre-training. The GCN series accelerates the processing with binarized descriptors and nested metric learning with groundtruths provided by the traditional Shi–Tomasi detector. Unsuperpoint [26] inherits the general idea of Superpoint, but relieves the reliance on labeled data by making the keypoint detection and matching totally self-supervised. However, its adaptability in the visual mapping process is unexplored.

### 2.2. Monocular Visual Architecture

Depending on the tasks defined, we review monocular visual architecture in terms of two main aspects, i.e., SLAM and SfM approaches. A milestone work of visual SLAM is the PTAM [27], which proposes an architecture of two parallel threads with one for motion tracking and the other for 3D feature-point mapping. However, suffering from the lack of loop closure and the low invariance to viewpoint change, its performance is limited in large environments and rapidly varying scenes. Built on the main idea of PTAM, ORBSLAM [28] utilizes ORB feature points through the pipeline of tracking, mapping, relocalization, and loop closing. It manifests an outstanding real-time performance and a good adaptability to RGB-D [29] and fisheye cameras [30]. However, the poor performance of ORB feature points in low-texture environments limits the application of ORBSLAM. VINS [31] adopts a more lightweight front-end with Harris corner detection and optical flow tracking. It also introduces an inertial sensor to minimize the scale variance of monocular-vision-only SLAM. Further improvements are also proposed with sensor adaptability, dynamic environment, online calibration, and pose-graph reuse [32,33,34,35,36].

SfM aims to estimate the 3D point positions as well as the camera poses through motion. Unlike SLAM, it can be applied on disordered images and the incremental methods are widely used. An early work was proposed by [37], which uses a pipeline of feature-point extraction, matching, and iterative pose optimization. Despite the relatively complete reconstruction obtained, this method suffers from mismatching in repeated scenes and a low processing speed due to the large number of images. Thus, subsequent research has focused more on the improvement of accuracy and efficiency. For example, ASFM [38] proposes adaptive thresholds in model estimation with a contrario methodology. It achieves better reconstruction results compared to those based on fixed global thresholds, yet at the cost of decreased calculation efficiency. In the further work of COLMAP [39], a geometric verification and a sampling-based triangulation are adopted to improve the robustness and completeness of reconstruction. Moreover, the global optimization is only then performed when the model grows to a certain extent, greatly reducing the load of computing resources and thus increasing the speed.

### 2.3. Mapping under Adversarial Illumination

The mapping task in low-illuminated environments such as underground mainly rely on lidar or camera sensors. Thus, corresponding approaches can be divided into two branches, depending on the used sensor. Lidar-based approaches prefer line or plane features. As the range information can be directly obtained with laser measurements, the cumulative error is relatively low. However, high-definition lidar is costly for commercialization and the positioning is difficult in structures such as long, narrow tunnels. For visual mapping, the main challenge remains in the visual feature extraction from low-texture, dim environments. Reference [13] proposed an underground mapping by adopting semantic features of parking slots in conjunction with a geometric prior to improve the point-matching accuracy. Additionally, they placed visual fiducial markers on specific positions to create a robust constraint in the back-end optimization. Despite an improved mapping performance, their dependence on visual markers restricts the usage of their method. Reference [40] proposed a self-supervised approach to learn features from both the entire image and its subregions. This method can effectively match images across daytime and thus improve the location-related image-retrieval accuracy. Reference [11] proposed a SLAM approach in indoor parking lots based on surround-view images to increase the perception range. Additionally, they built maps by exploiting semantic segmentation results of parking signs, slot lines, and bumps as robust features against illumination change, achieving a centimeter-level accuracy. Reference [41] proposed CNN models to accomplish the identification of slot marking points and classification of patterns represented by marking-point pairs that can be applied on both indoor and outdoor parking sites. Reference [42] proposed a more lightweight parking-slot segmentation model by employing network pruning. Their method shows a significantly reduced computational cost and can be applied on CPUs with real-time performance while maintaining a good mapping precision. Nevertheless, the segmentation performance strongly relies on the quantity and quality of pixel-wise labeled data, which restricts the generalizability of the above mapping approaches.

In this work, we mainly focus on the mapping accuracy. Thus, without strict runtime requirements, we adopt an SfM scheme, i.e., the COLMAP, as the back-end for camera pose optimization. The proposed approach is presented mainly based on the mapping task of an underground parking lot. To improve the mapping performance, we adopt an unsupervised learning method to address the challenge of feature-point detection and -description in dim, low-texture environments. Moreover, we integrate the multi-grained image similarity measurement along with feature-point verification into the loop-closure detection and adopt ground-point features with camera height for scale recovery, thus improving the mapping robustness.

## 3. Proposed Approach

**Figure 2:**
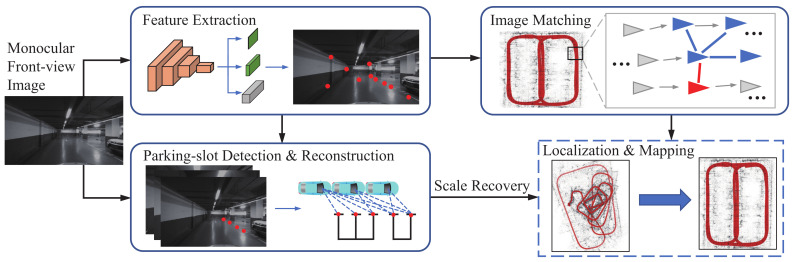
The overall architecture of our monocular visual mapping system.

The pipeline of the proposed visual mapping approach is shown in Figure 2. The input image is captured with a monocular front-view camera on a vehicle and fed into two subsequent processing branches. The first branch adopts a CNN to predict the scores, relative positions, and descriptors of the keypoints, which are learned in an unsupervised manner. The keypoints are matched across frames and fed into the SfM framework along with the corresponding image pairs to reconstruct a scene graph for camera pose estimation. In conjunction with the keypoint-based verification, it also adopts a multi-grained image similarity measurement to improve the pose-estimation accuracy, especially for loop closures. In the second branch, it detects the corners of ground parking slots and considers them as additional keypoints, which are tracked across frames by leveraging the keypoint network in the first branch. By integrating the tracks of the above-ground keypoints and the camera height, the absolute scale of camera motion can be recovered. With an additional association strategy, the parking slots on the ground can be reconstructed and thus the map for the underground parking lot can be created. The specific steps in this proposed mapping procedure are introduced below.

### 3.1. Unsupervised Keypoint Extraction

**Figure 3:**
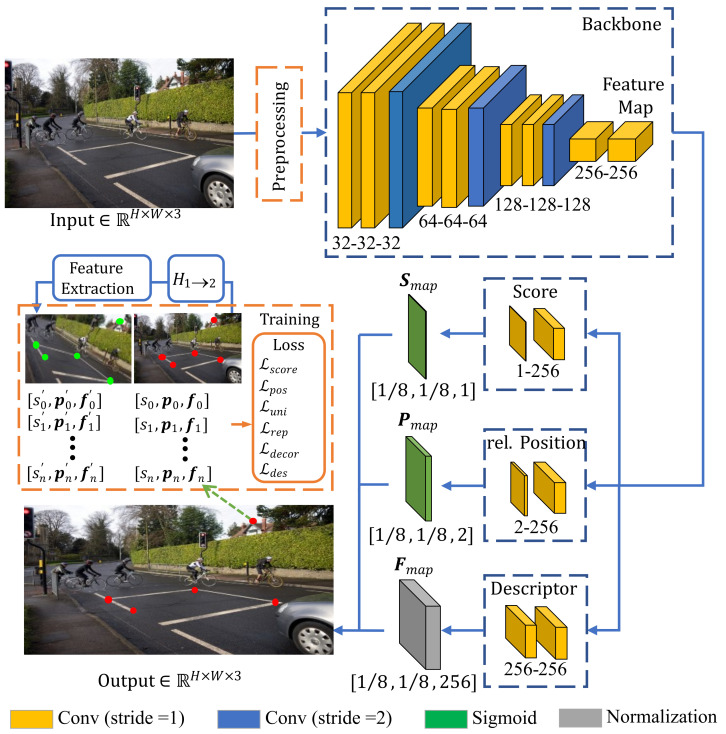
The architecture of our feature-extraction network. The channel number is indicated under each block. The network takes an input image and outputs a feature-point vector (s,p,f).

Regarding both the scene adaptability and the unreliability on training labels, in this work, we adopt an unsupervised-learning-based approach for keypoint extraction. The network consists of a backbone similar to reference [26] except with more convolutional layers to facilitate the learning of deep visual features. We additionally enhance the loss function by emphasizing the consistence between feature points in illumination-varied environments. The detailed architecture of the network is shown in Figure 3.

The input image is first processed by the backbone (top right in Figure 3), which consists of four stages. After each stage, the feature map is downsampled by a factor of 2 while its channel number is doubled. The generated feature map thus has a size of 1/8 of the input image and is further fed into three output heads to predict the tensor of keypoint scores $S_{map}$, relative positions $P_{map}$, and descriptors $F_{map}$, respectively. Since the output tensors are the same size as the input feature map, the total predicted keypoints is $\frac{H}{8}\times \frac{W}{8}$, and each score, relative position, and descriptor correspond to an 8 × 8 region of the input image.

For training, a source image $I_{1}$ is preprocessed using a random homography transform $H_{1\to 2}$ with an additional color conversion or noising to generate a warped version $I_{2}$ (see orange dashed box in Figure 3). Thus, we obtain an image pair ($I_{1}$, $I_{2}$), which is fed into the network to predict keypoints. Each keypoint P consists of a tuple of score, relative position, and descriptor, described as (s,p,f). Similar to reference [26], descriptor f is a 256-dimensional tensor of floating-point numbers. A keypoint $P_{1,i}$ in $I_{1}$ with another keypoint $P_{2,j}$ in $I_{2}$ is defined as a good match if their Euclidean distance $∥H_{1\to 2}\cdot p_{1,i}-p_{2,j}{∥}_{2}$ is less than a threshold $\alpha_{dis}$.

The loss function of the whole network consists of six terms as

(1) $$\begin{array}{cc} L_{total}= & w_{1}L_{score}+w_{2}L_{pos}+w_{3}L_{rep}+w_{4}L_{uni}+w_{5}L_{decor}+w_{6}L_{des}, \end{array}$$

where $w_{1,\ldots ,6}$ indicates the corresponding weight of each term. The losses $L_{score}$, $L_{pos}$, $L_{rep}$, $L_{uni}$, and $L_{decor}$ are defined similar to reference [26] and briefly described in Table 1 and Appendix A.1.

The essence of a keypoint description is the expression of a corresponding image patch surrounding the point. Considering that similar image areas should yield highly correlated keypoint descriptors, the descriptor loss $L_{des}$ should reduce their distance in the feature space. Inspired by the contrastive learning of visual representation [43], the loss $L_{des}$ is defined as

(2) $$L_{des}=\sum_{i}^{}-\log\frac{\sum_{j}^{}\exp(t\cdot f_{1,i}{}^{⊤}\cdot f_{2,j})}{\sum_{l\neq j}\exp(t\cdot f_{1,i}{}^{⊤}\cdot f_{2,l})}$$

where terms $\left(f_{1,i},f_{2,j}\right)$ in the nominator indicate keypoint descriptors of a good match, while keypoint descriptors $\left(f_{1,i},f_{2,l}\right){|}_{l\neq j}$ in the denominator are from a bad match. The temperature *t* is a hyperparameter. For hard negatives, which can be easily classified as false positives, a smaller *t* can reduce their weights during learning, which further improves the feature-point matching.

In order to improve the illumination robustness of keypoints, we utilize traditional transformation methods such as random clipping, flipping, and brightness adjustment, with the latter having a certain impact on the performance of feature points in adversarial-illumination environments. Moreover, our adopted loss function $L_{des}$ contributes to improving the feature-point learning significantly. Detailed experimental verification can be seen in Section 4.2.

### 3.2. 3D Reconstruction with Improved Scale Estimation

The predicted image keypoints are tracked across frames (e.g., by descriptor matching) and then fed into the 3D reconstruction framework. Here, we use COLMAP [39] as the base reconstruction approach. Additionally, we add the multi-grained image matching along with the feature-point verification to reduce drift at loops and adopt ground keypoints for further scale recovery.

#### 3.2.1. Base SfM Approach

COLMAP is an incremental SfM framework. Given a set of images with correspondences indicated by tracked keypoints, also known as the scene graph, the outputs are the estimated poses of camera images and the scene structure represented by a point cloud in the 3D space. For initialization, it uses a seeding with an elaborated two-view reconstruction, e.g., at a location in the image graph with multiple overlapping cameras, which has a high redundancy. It further proceeds with incremental registration of new images. Their poses are estimated by solving the Perspective-n-Point (PnP) problem based on the triangulation of matched feature points in exiting images. By extending the camera pose, new scene points can be observed and triangulated from the added images, thus incrementally increasing the scene coverage. To reduce the uncertainties propagated between the image pose and triangulated points, bundle adjustment (BA) is utilized as a joint non-linear refinement for both of them by minimizing the point reprojection errors on the image. Additionally, COLMAP leverages the multi-model to verify non-panoramic and calibrated image pairs in seeding the reconstruction. To reduce the risk of mis-registration, it chooses the next-best view based on a multi-resolution analysis. Furthermore, it adopts an efficient sampling-based triangulation and an elaborated local/global BA strategy. Although COLMAP has improved over conventional SfM approaches in the reconstruction completeness and scalability, it still shows limitations when applied to monocular images. Lacking the depth information, the scale estimation of a monocular SfM system significantly depends on the feature points in the local map. Due to the inherent uncertainties of feature points (e.g., by misdetection and mismatching), without sufficient constraints, it is difficult to maintain a long-term scale consistency and thus makes the monocular system susceptible to scale drifts. Therefore, more powerful constraints such as loop-closure detection (which is common in parking lots and urban streets) are required so that the scale information of feature points can be efficiently propagated.

#### 3.2.2. Image Matching with Multi-Grained Similarity and Keypoint Verification

Compared to keypoint descriptors, images contain much richer visual information. Thus, we adopt an image-retrieval-based loop-closure detection, i.e., to identify images captured at the same place. However, spatially close-by images do not exactly depict the same scene, especially with varied camera poses or changed foreground objects, which results in noisy hard positives. By only using the image-level supervision, all the features of the target image are forced to be similar to those of the noisy positive image, impairing the discriminatory learning of local features. Regarding these points, we employ a multi-grained image-similarity learning strategy. Specifically, we decompose each image into 4 half-regions (including horizontal and vertical direction) and 4 quarter-regions. Thus, the similarity of the target and the positive image is learned in a region-level supervision. Compared to the image-level supervision, such a manner is more rational. It focuses more on the common regions between images during learning by pulling positive regions closer while pushing away negative regions in the feature space.

Here we adopt a VGG16-based network as in [40], which receives the query and noisy positive image as inputs and estimates their similarities. Additionally, we adopt an iterative learning strategy, i.e., using the converged model and its estimated image similarities in the current round to initialize the to-be-trained model and its supervised labels in the next round. The positive inputs are the top *k* (k=10) difficult images with their queries from the last round. By iteratively mining the hard positives, the accuracy of the predicted similarity labels and the discriminatory ability of the model are progressively improved in a self-supervised manner. The loss function is defined as in [40], which is

(3) $$L=L_{hard}+\lambda L_{soft},$$

where λ is a trade-off factor. The first term is in the form of

(4) $$L_{hard}=\sum_{j}\log\frac{\exp(f{v_{q}}^{⊤}\cdot fv_{p})}{\exp\left(f{v_{q}}^{⊤}\cdot fv_{p}\right)+\exp\left(f{v_{q}}^{⊤}\cdot fv_{n_{j}}\right)},$$

where $f_{v}$ indicates the feature vector extracted from the last convolutional layer of VGG16 (i.e., the conv5) while the subscripts *q*, *p*, and $n_{j}$ denote the query, the positive, and the hard negative *j*, respectively. The second loss term in Equation (3) is expressed as

(5) $$L_{soft}=\mathrm{CE}\left(s\left(q,p_{1}^{r},\ldots ,p_{n}^{r},1\right),s_{-1}\left(q,p_{1}^{r},\ldots ,p_{n}^{r},\tau \right)\right)$$

with CE denoting the cross entropy operator. The subscript “−1” indicates features extracted from the model trained in the previous round. The temperature τ is a hyperparameter. The similarity vector is calculated in a softmax form as

(6) $$s\left(q,p_{1}^{r},\ldots ,p_{n}^{r},\tau \right)=\mathrm{softmax}\left(\frac{f{v_{q}}^{⊤}\cdot fv_{p_{1}^{r}}}{\tau },\ldots ,\frac{f{v_{q}}^{⊤}\cdot fv_{p_{n}^{r}}}{\tau }\right),$$

where $p_{1}^{r},\ldots ,p_{n}^{r}$ indicate the top *k* hard positive images and their 8 subregions. Thus, we have the number n=9k. In our experiment, the network is trained in 4 rounds with progressively reduced τ (0.07, 0.06, 0.05), while the trade-off factor is set to λ=0.5.

Given the fact that our keypoint network can extract an amount of robust keypoints and all images are acquired consecutively, we adopt a sequential image-matching procedure in conjunction with the loop-closure detection to suppress the uncertainty of scales. The image matching is based on the multi-grained similarity measurement and an additional feature-point verification, with the latter to suppress the false positives missed by the former. The whole image-matching process is as follows.

**Figure 4:**
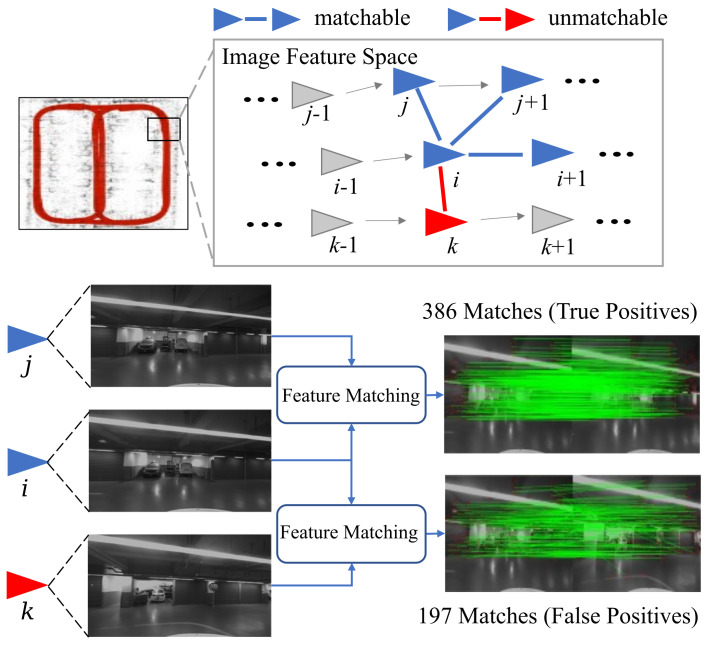
The sequential image-matching process.

1. Images are processed in chronological order. If there is still an image unprocessed, it is marked as $q_{i}$. Otherwise, the process is terminated.
2. The query $q_{i}$ is matched with its following *N* images.
3. Based on the matching results, the images are searched with their multi-grained image similarities to $q_{i}$ within a threshold $\alpha_{mg}$ and denoted as set O. For an empty O, the procedure goes back to step 1.
4. For each image $p_{j}\in O$, if the number of correspondence keypoints between $q_{i}$ and $p_{j}$ is greater than a threshold $\alpha_{num}$, the pair $\left(q_{i},p_{j}\right)$ is recorded into a database of query-positive candidates (Figure 4). Otherwise, the procedure goes back to step 1.
5. For each $p_{j}$ in the candidate database, we also consider the correspondence between $q_{i}$ and *N* images after $p_{j}$. If the number of correspondence keypoints in any image pair is not greater than the threshold $\alpha_{num}$, the candidate database is cleared. The assumption is that the keypoints across true positive images can be tracked for a period. Otherwise, the candidate database is recorded in the final database. The procedure goes back to step 1.

In the above process, we empirically set N=3, $\alpha_{mg}=0.5$, and $\alpha_{num}=250$.

#### 3.2.3. Scale Recovery

This part focuses on the reconstruction of underground parking slot instances and the recovery of the overall scale in the mapping task.

**Figure 5:**
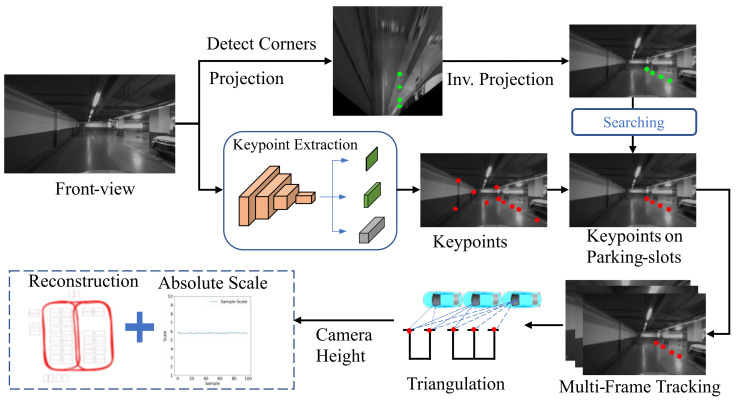
The process for scale recovery and parking slot instance reconstruction.

Since the corners of parking slots appear to be similar and can be easily recognized in the bird’s eye view (BEV), we first convert the front-view image to a BEV image using the process introduced in reference [44] and use a CenterNet-based network [45] to detect the corner points of parking slots. Afterwards, we project the positions of the detected corner points from the BEV back to the front-view image. The feature points related to the projected corner points are searched and tracked in the next few frames. As the corner points are strong keypoints, they can provide a more stable tracking performance. The related procedure is shown in Figure 5.

Due to the restricted driving speed, each parking slot as well as its corners can be observed in up to 20 consecutive frames. With triangulation, we can obtain the positions of the corner points in the 3D space. Since each parking slot has two corresponding corner points at the entrance, the position of the parking slot (represented by the entrance center) can be estimatedwithby two corner points within a distance threshold (i.e., 2.5 m).

**Figure 6:**
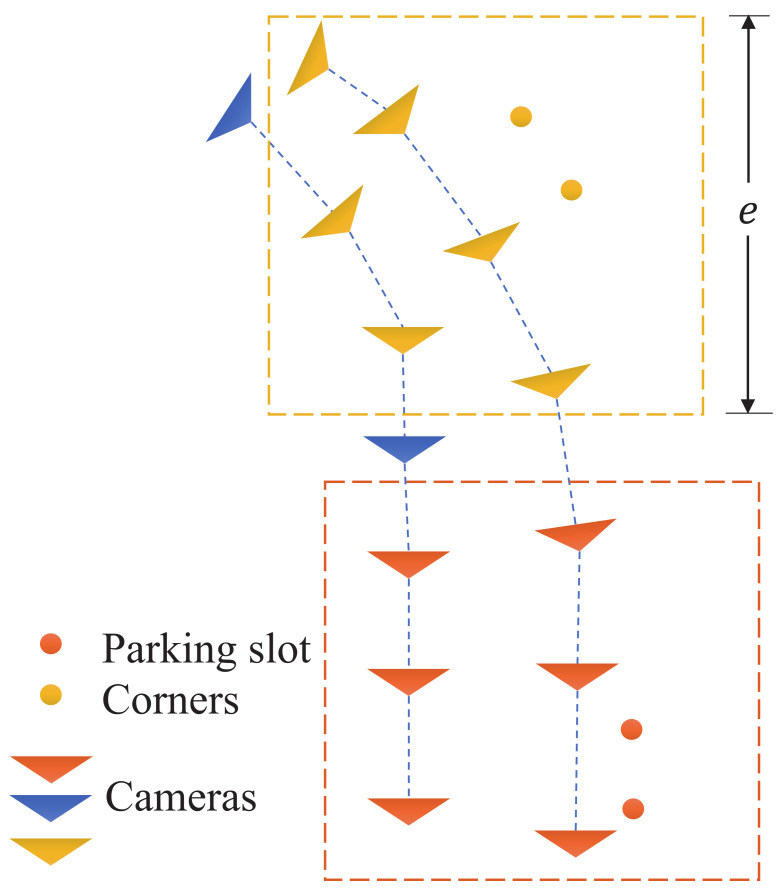
Local scale recovery based on selected camera trajectories.

The scale recovery is based on the relative distance between the ground and the camera trajectory. Specifically, for each pair of corner points, we consider a 3D cubic space located at the center of the corner points, as illustrated in Figure 6. The cube is above the ground plane with a side length of *e* greater than the camera height *d*. Given a camera position t, the ground plane can be approximated by

(7) $$n^{⊤}t+d=0,$$

where n indicates the plane normal vector. Given a camera trajectory within the cube, its historical positions $\left(t_{1},t_{2},\ldots ,t_{k}\right)$ can be applied with the above equation as

(8) $$\left[\begin{array}{c} t_{1}^{⊤}\\ t_{2}^{⊤}\\ \vdots \\ t_{k}^{⊤} \end{array}\right]\cdot n+d=0.$$

Here we assume that the ground is flat and the camera height is fixed within the cube. The cube size is elaborately set so that it contains sufficient trajectory points and Equation (8) is over-determined. Thus, the normal vector n and the camera height *d* can be solved, e.g., using the least squares method. Given the real measurement $d_{gt}$ of the camera height, the scale correction factor is $r=\frac{d_{gt}}{d}$, which is used to recover the scale of the reconstructed map, especially the parking slot instances. The math symbols used in our paper are explained in Table A1 in Appendix A.2.

## 4. Experiment

### 4.1. Platform Configuration and Dataset Selection

In our work, we implement the keypoint extraction network and the mapping approach with Python 3.6.9, PyTorch 1.7.0 on Ubuntu 18.04 LTS using a machine with 32 GB RAM, a six-core Intel Core i7-8700 CPU, and an Nvidia GeForce GTX 1060 GPU.

For the keypoint extraction learning, we util-ze MS COCO [46] to train the network and test it on HPatches [47]. For the evaluation of the 3D reconstruction and mapping, we test our approach on EuRoC [17] and KITTI [18]. In comparison with other unused datasets such as Strecha [48] and DTU [49], the datasets selected here are more comprehensive in terms of the data amount and diversity, up-to-date, and broadly used to validate mainstream methods, which allows a fair comparison of our method with state of the art.

### 4.2. Evaluation of Keypoint Model

For a comprehensive evaluation of the utilized keypoint detector, it is tested on the HPatches benchmark [47], which consists of sequences from 59 scenes with viewpoint change and 57 scenes with illumination variation, each with one reference image and a number of target images. All images are scaled to the same size of 240 × 320 px for evaluation. Four metrics are chosen, i.e., the homography accuracy (HA), the repeatability score (RS), the location error (LE), and the matching score (MS), which are briefly described in Table 2. The loss weights are empirically set to $\omega_{1}=2$, $\omega_{2}=1$, $\omega_{3}=1$, $\omega_{4}=100$, $\omega_{5}=0.03$, and $\omega_{6}=0.001$ (with t=0.05). It is trained on the MS COCO dataset [46] for 10 epochs with a learning rate of 0.000025 and a batch size of 16. The ADAM optimizer is adopted. The inference time of our keypoint model is about 10 ms per image. The test results of the utilized keypoint model on scenes with illumination variation and viewpoint change are reported in Table 3, in comparison with handcrafted feature points such as SIFT and SURF and another self-supervision-based method, Superpoint.

**Figure 7:**
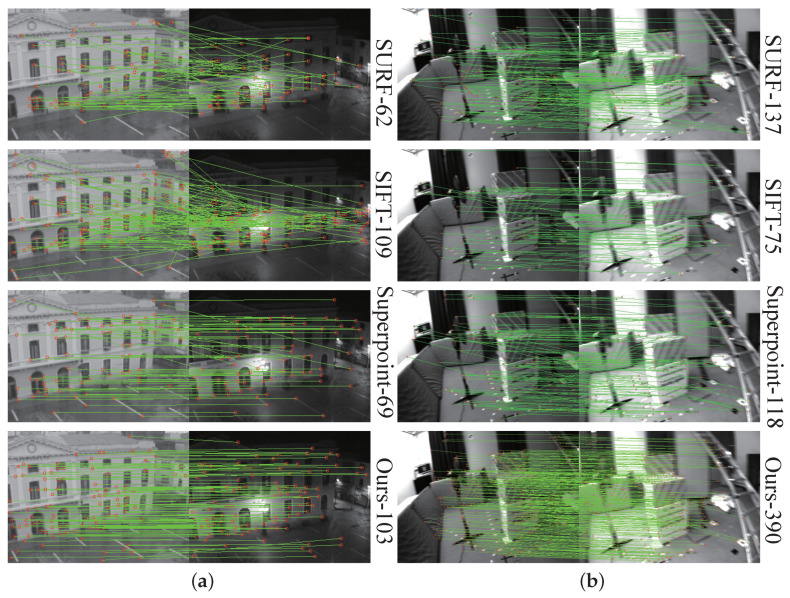
Qualitative comparison of matched keypoints using different models on scenes with (**a**) illumination change and (**b**) viewpoint change. Detected keypoint number is displayed on the right side of each figure.

As can be seen, the learning-based keypoint models significantly outperform the handcrafted approaches in handling scenarios with illumination variation. Especially in terms of the matching score, the gain is more significant, up to 38%, implying a more powerful feature representation with deep learning. Moreover, the adopted keypoint model in our approach achieves a slightly lower HA than Superpoint while outperforming it in terms of all three other metrics. The improvement in the location error is more critical, at 13%, demonstrating the effectiveness of unsupervised learning of the keypoint detector. A qualitative example is shown in Figure 7a. As for scenes with a viewpoint change, similar trends can be seen in Figure 7b. Deep-learning-based approaches achieve a comparable HA to SIFT while outperforming it in all other three metrics, with our adopted approach ranking at the top.

To assess the influence of brightness adjustment during preprocessing and the loss term $L_{des}$ on the illumination robustness of the learned feature points, we conducted ablation experiments on the HPatches illumination sequence, with the results presented in Table 4. The results indicate that the performance of feature points is influenced by both traditional transformations and $L_{des}$. Only by combining these factors can the optimal performance of feature points be achieved.

### 4.3. Study on Image Matching and Keypoint Verification

To verify the performance of the adopted multi-grained image matching and keypoint verification, we collected a dataset by driving a test vehicle for several rounds in the underground parking lot of about 1500 $m^{2}$ at Tongji University Campus. The test vehicle was equipped with a camera capturing front-view images at 10 Hz and a lidar of 16 beam lines. As GNSS signals are unavailable in the underground space, we consider the map constructed using the laser measurements of lidar as the groundtruth. An ablation study on the influence of the adopted multi-grained image matching and keypoint verification was conducted, with the results reported in Table 5. For evaluation, it follows the protocol of the 7-DOF alignment [50] of the reconstructed trajectories. The mean value of absolute pose errors (APE) is chosen as the metric.

**Figure 8:**
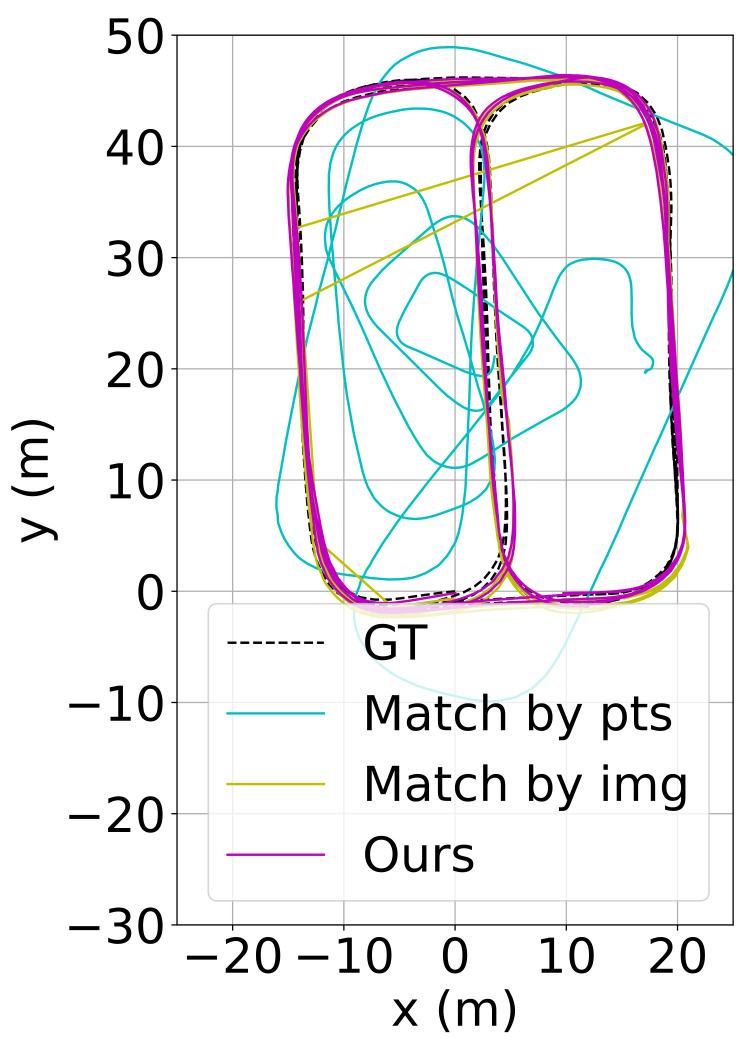
The estimated trajectories using different matching schemes.

It is straightforward that the naive matching by keypoints yields a high position error of more than 12 m. By replacing it with the multi-grained image matching, the error is reduced to 0.6 m. However, image mismatching still occurs, which leads to an abrupt change in the estimated trajectory (yellow curves in Figure 8). By further integrating the keypoint verification procedure, the error decreases to 0.45 m and the abrupt trajectory change disappears (purple curves in Figure 8). Thus, the benefit brought by multi-grained image matching and keypoint verification has been validated.

### 4.4. Exploration of Mapping Architecture

**Figure 9:**
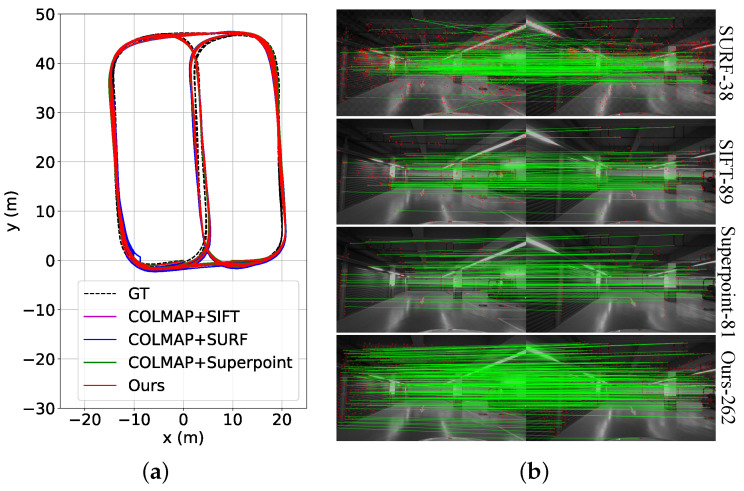
(**a**) The mapping results using different approaches. (**b**) Matched keypoints using different approaches.

**Figure 10:**
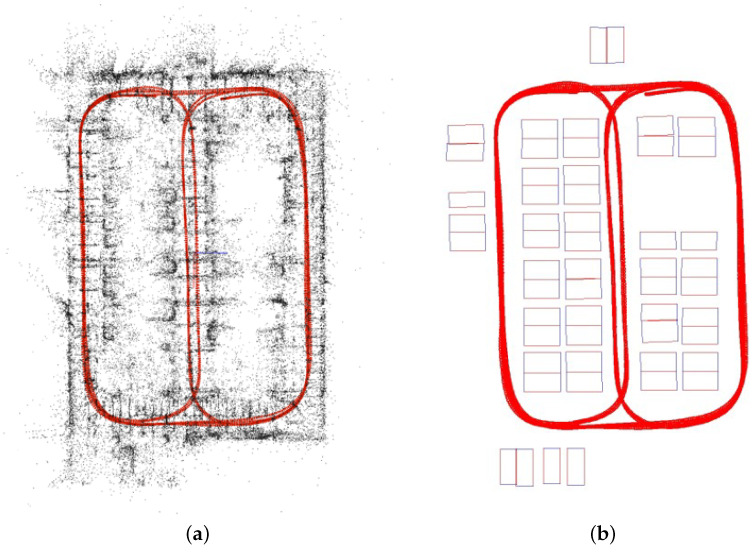
(**a**) Generated map in representation of point clouds. (**b**) Generated map with parking slot identification.

Although the keypoint model alone was tested in the previous experiments, its influence on the SfM-based mapping architecture is still unexplored. Here we replace our adopted keypoint model with other approaches such as the handcrafted SIFT and ORB and the learning-based Superpoint, thus yielding three new mapping architectures, denoted *COLMAP (SIFT)*, *COLMAP (SURF)*, and *COLMAP (Superpoint)*. We adopt the same data and metrics as in Section 4.3, with the test results shown in Table 6. Obviously, the learning approach of Superpoint improved the mapping accuracy compared to the handcrafted SIFT and SURF in terms of the mean APE. Since our keypoint model emphasizes the distinctive feature representation during training, by integrating it, the least pose error has been achieved. The estimated trajectories and keypoint matches are illustrated in Figure 9. Additionally, we show the map generated by our approach in representation of point clouds and with identified parking slots in Figure 10a,b, respectively.

### 4.5. Transferring on SLAM Approaches

Although our matching scheme is designed for SfM architectures, it can be integrated with SLAM systems. Here we replace COLMAP with the VINS approach. Due to the high complexity of the optimization scheme in VINS, we exclude the integration of multi-grained image matching and keypoint verification. We test the new architecture on the EuRoC and KITTI datasets. The EuRoC dataset is aimed for the evaluation of indoor SLAM and consists of 11 sequences captured in different rooms and fields along with IMU measurements. The KITTI visual odometry dataset is captured in a surrounding region of the city Karlsruhe, consisting of 22 image sequences, in which 11 sequences (00-11) are provided with temporally aligned groundtruths and measurements from lidar sensors. For comparison, we provide the performance of the original VINS approach, which is denoted as *VINS (flow)* due to its deployment of optical flow. Additionally, we replace the optical flow in VINS with other feature descriptors and our proposed feature-point approach and report their test performance. The evaluation metric is the root mean square error (RMSE) of the estimated trajectories.

#### 4.5.1. Evaluation on EuRoC

**Figure 11:**
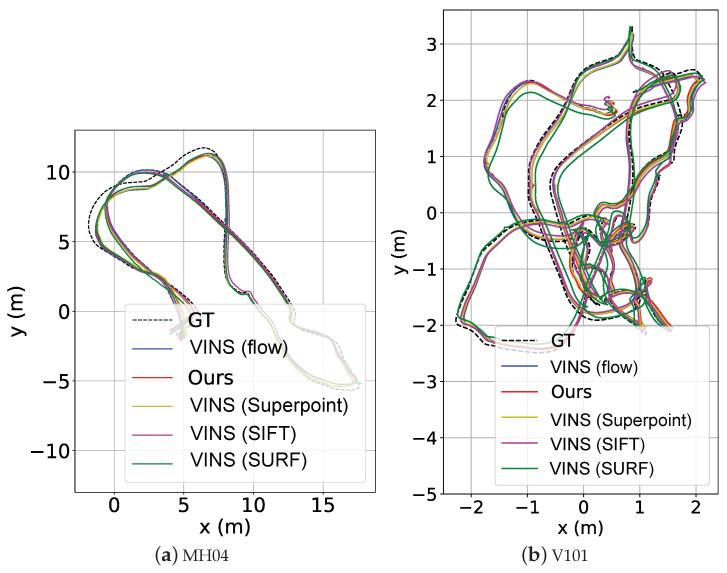
Estimated trajectories in sequence MH04 and V101 on EuRoC.

The evaluation results are shown in Table 7 while examples of the estimated trajectories in the sequence MH04 and V101 are shown in Figure 11. As can be seen, the handcrafted feature points generally perform inferiorly to the learning-based approaches. Specifically, the *VINS (flow)* employs the conventional Harris keypoint detector with its point tracking assisted by optical flow. Its accuracy is still lower than our approach and the version with Superpoint. Superpoint dominates mainly on the “M”-sequences, which have rich textures, benefiting the accuracy and stability of the keypoints extracted by Superpoint. In contrast, our approach is more advantageous on the “V”-sequences with large camera motion and illumination variation. The adverse environmental conditions can result in reduced keypoints extracted by other approaches. However, as our method focuses more on the learning of distinctive visual features, it shows a superior performance. An example of feature-point matching with large camera motion is visualized in Figure 7b.

#### 4.5.2. Evaluation on KITTI

**Figure 12:**
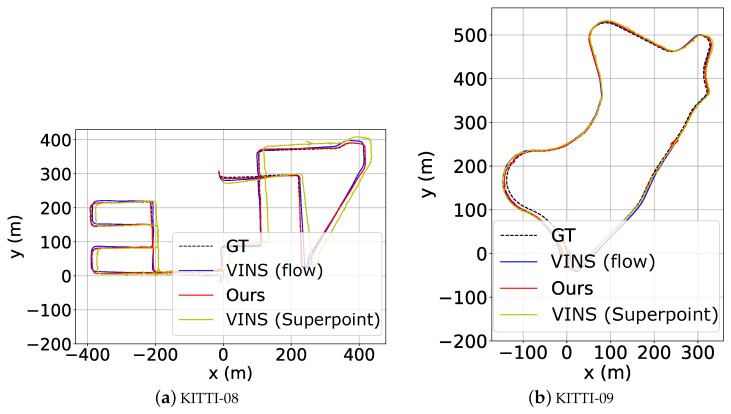
Qualitative test results on sequence KITTI08 and KITTI09.

Due to the fast motion in large-scale outdoor environments, the VINS versions integrated with handcrafted keypoint approaches such as SIFT and SURF all fail in the test, thus without the results reported in Table 8. The high-speed motion scenario (e.g., the highway) also explains the failure of learning-based approaches in sequence 01. Since *VINS (flow)* employs optical flow to assist the point tracking, it can still maintain a small position error in this sequence. Furthermore, it can be seen that our approach dominates the sequences 00, 02, 05, and 08–10 with the least error while in other sequences it performs better than Superpoint, yet with a small gap to *VINS (flow)*. Especially in sequences 00, 02, and 08, our approach outperforms *VINS (flow)* more significantly, with an accuracy gain of more than 4 m, further demonstrating the effectiveness of our approach. Examples of estimated trajectories are shown in Figure 12.

## 5. Conclusions

In this paper, we proposed a novel monocular visual-mapping approach to address adversarial illumination conditions such as in underground parking lots. There are two main points contributing to this approach: the unsupervised learning of keypoints that enhances the discriminative feature representation and the scheme of multi-grained image matching and keypoint verification in scale drift suppression. This paper presents extensive experiments validating the individual modules and the entire SfM-based mapping architecture, demonstrating the robustness of the approach against illumination variation. The testing results on a public benchmark and our collected dataset show that our approach outperforms the existing mainstream methods, with an average location error gap of 0.05 m on collected data, 0.04 m on Euroc, and 1.86 m on KITTI. Additionally, the approach is effective not only in SfM systems but also in indoor SLAM and outdoor visual odometry tasks.

## Figures and Tables

**Table 1:** Loss terms to train keypoint network.

Term	Description
$L_{score}$	Squared score difference of paired points
$L_{pos}$	Euclidean distance of paired points
$L_{uni}$	Differences between the distribution of predicted point coordinates and a uniform distribution
$L_{decor}$	Correlation coefficients between keypoint descriptors on the same image, further explained in Equation (A.1)
$L_{rep}$	Ensuring closely located point pair with a high score, interpreted as $L_{rep}=\sum_{k}\left(s_{1}+s_{2}\right)\left(d_{k}-\bar{d}\right),$ with $s_{1},s_{2}$: point scores of the *k*-th pair $d_{k}$: distance of the *k*-th pair $\bar{d}$: mean distance of all pairs

**Table 2:** Evaluation metrics of the keypoint network.

Metric	Description
HA	Ratio of estimated homographies under a threshold ϵ (here set to 3 px) to all estimated homographies
RS	Ratio of corresponding points to all predicted points
LE	Average distance of corresponding points
MS	Ratio of good matches to predicted points in one image, where a good match denotes two corresponding points with the nearest descriptors in the feature space.

**Table 3:** Comparison of different keypoint detectors on the sequence with viewpoint and illumination change of HPatches.

	Illumination				Viewpoint			
Methods	HA ↑	RS ↑	LE ↓	MS ↑	HA ↑	RS ↑	LE ↓	MS ↑
SURF	0.77	0.57	1.16	0.27	0.58	0.53	1.41	0.23
SIFT	0.86	0.50	1.11	0.25	**0.66**	0.52	1.22	0.29
Superpoint	**0.93**	0.64	0.94	0.63	0.63	0.51	1.17	**0.47**
Ours	0.91	**0.65**	**0.81**	**0.64**	0.62	**0.55**	**1.09**	**0.47**

**Table 4:** Ablation study results on HPatches illumination sequence. “Trans.” indicates brightness transformations and “$L_{des}$” indicates the utilization of the loss function $L_{des}$.

Trans.	$L_{\mathrm{des}}$	HA ↑	RS ↑	LE ↓	MS ↑
✓		0.86	0.65	0.82	0.38
	✓	**0.91**	0.65	0.92	0.62
✓	✓	**0.91**	0.65	**0.81**	**0.64**

**Table 5:** Ablation study results. “Match by pts.” indicates the naive matching by keypoints; “Match by img.” indicates the multi-grained image matching; “Pts. verify” indicates the keypoint verification procedure; “-as” represents the 7-DOF alignments used in the evaluation.

Match	Match	Pts.	APE-as
by Pts.	by Img.	Verify	(Mean) ↓
✓			12.81 m
	✓		0.60 m
	✓	✓	**0.45 m**

**Table 6:** Exploration of SfM-based mapping integrated with different keypoint models.

Methods	APE-as (Mean) ↓
Ours	**0.453 m**
COLMAP (Superpoint)	0.502 m
COLMAP (SIFT)	0.562 m
COLMAP (SURF)	0.596 m

**Table 7:** The RMSE (in meters) of estimated trajectories using VINS integrated with different keypoint models on EuRoC. “x” indicates the failure of approach.

	VINS		VINS	VINS	VINS
Seq.	(Flow)	Ours	(Superpoint)	(SIFT)	(SURF)
MH01	0.24	0.22	**0.20**	0.76	0.50
MH02	0.22	0.22	**0.18**	0.51	0.48
MH03	0.28	0.24	**0.17**	x	0.27
MH04	0.43	**0.43**	0.47	0.56	0.62
MH05	0.31	0.32	**0.22**	0.53	0.75
V101	0.109	**0.108**	0.12	0.23	0.20
V102	0.10	0.11	**0.09**	0.13	0.13
V103	0.111	**0.088**	0.09	0.15	0.19
V201	0.121	**0.116**	0.14	0.18	0.21
V202	0.11	**0.09**	0.13	0.27	0.25
V203	0.30	**0.20**	0.79	0.39	0.49

**Table 8:** The RMSE (in meters) of estimated trajectories using VINS integrated with different keypoint models on KITTI. “x” indicates the failure of approach.

	VINS		VINS
Seq.	(Flow)	Ours	(Superpoint)
00	13.74	**7.05**	77.70
01	**7.54**	x	x
02	20.69	**11.34**	17.71
03	**1.75**	2.67	3.17
04	**1.33**	2.27	2.68
05	6.64	**5.54**	5.78
06	**3.87**	4.89	15.64
07	**2.20**	4.27	11.28
08	9.37	**4.97**	57.24
09	7.73	**7.42**	8.01
10	3.66	**1.96**	4.25

## Data Availability

The datasets generated and analysed during the current study are available in the [KITTI] repository and the [EuRoC] repository. (http://www.cvlibs.net/datasets/kitti (accessed on 9 February 2023)) and (https://projects.asl.ethz.ch/datasets/doku.php?id=kmavvisualinertialdatasets (accessed on 9 February 2023)).

## Appendix A. Mathematical Explanations

### Appendix A.1. Equation of $L_{decor}$

Descriptors should be decorrelated to prevent overfitting and carry as much useful information as possible within a limited dimension. The descriptors forming good matches in image pair ($I_{1}$, $I_{2}$) are denoted as $G_{1}={\left[f_{1}^{1},\ldots ,f_{1}^{m},\ldots ,f_{1}^{p}\right]}_{256\times p}$ and $G_{2}={\left[f_{2}^{1},\ldots ,f_{2}^{m},\ldots ,f_{2}^{p}\right]}_{256\times p}$, where *p* stands for the number of good matches. Apparently, we can denote $G_{1}{}^{⊤}$ as ${\left[h_{1}^{1},\ldots ,h_{1}^{m},\ldots ,h_{1}^{256}\right]}_{p\times 256}$ and $h_{1}^{m}{}^{⊤}$ is the *m*-th row vector of $G_{1}$. Similar to reference [26], the correlation matrix $R_{1}={\left[r_{ij}^{1}\right]}_{256\times 256}$ can be defined as

(A1) $$r_{ij}^{1}=\frac{\left(h_{1}^{i}-{\bar{h}}_{1}^{i}\right){}^{⊤}\left(h_{1}^{j}-{\bar{h}}_{1}^{j}\right)}{\sqrt{\left(h_{1}^{i}-{\bar{h}}_{1}^{i}\right){}^{⊤}\left(h_{1}^{i}-{\bar{h}}_{1}^{i}\right)}\sqrt{\left(h_{1}^{j}-{\bar{h}}_{1}^{j}\right){}^{⊤}\left(h_{1}^{j}-{\bar{h}}_{1}^{j}\right)}}$$

where ${\bar{h}}_{1}^{i}$ stands for the mean of the *i*-th row of $G_{1}$. The same is true for $R_{2}$. Thus, we can calculate $L_{decor}$ as

(A2) $$L_{decor}=\sum_{i\neq j}{\left(r_{ij}^{1}\right)}^{2}+\sum_{i\neq j}{\left(r_{ij}^{2}\right)}^{2}.$$

### Appendix A.2. Explanation for Math Symbols

**Table A1:** A notation table that explains math symbols in our used paper.

Symbol	Description
$S_{map}$	Map of keypoint score
$P_{map}$	Map of keypoint relative positions
$F_{map}$	Map of keypoint descriptors
H,W	Height/Width of input images
$I_{1},I_{2}$	Source image/Warped image
$H_{1\to 2}$	Homography transform matrix
P	Keypoint
*s*	Keypoint score
p	Keypoint relative position
f	Keypoint descriptor
L	Loss function
$w_{1,\ldots ,6}$	Weights of losses
*t*	Hyperparameter that controls $L_{des}$
λ	Trade-off factor between $L_{hard}$ and $L_{soft}$
$f_{v}$	Feature vector
s	Similarity vector
τ	Hyperparameter that controls $f_{v}$ and s
$q_{i}$	Unprocessed image
*N*	Number of images following $q_{i}$
O	Set of images that are not searched
$p_{j}$	Image that is not searched
$\alpha_{mg}$	Threshold of multi-grained image similarities
$\alpha_{num}$	Threshold of the number of correspondence keypoints
*d*	Camera height
*e*	Side length of the cubic space in Figure 6
t	Camera position
n	Plane normal vector
*r*	Scale correction factor
